# Why the Chosen Ones May Not Always Be the Best Leaders: Criteria for Captain Selection as Predictors of Leadership Quality and Acceptance

**DOI:** 10.3389/fpsyg.2020.616966

**Published:** 2021-01-18

**Authors:** Radhika Butalia, Katrien Fransen, Pete Coffee, Jolien Laenens, Filip Boen

**Affiliations:** ^1^Department of Movement Sciences, KU Leuven, Leuven, Belgium; ^2^Faculty of Health Sciences and Sport, University of Stirling, Stirling, United Kingdom

**Keywords:** team captain, athlete leadership, peer leadership, captain selection, leadership quality, leader acceptance

## Abstract

There seems to be some initial evidence that team captains are selected based on non-leadership factors such as team tenure, technical abilities, being the daughter of the club president, or playing position. This is concerning since players expect their ideal team captain to have superior motivational and social skills. Adding to this literature on captain selection, the present study investigates relationships between the reasons for which team captains are selected and their (a) perceived leadership quality; and (b) perceived acceptance. To accomplish this, we recruited 450 coaches and 198 players from Flemish football and volleyball teams. Participants evaluated 41 reasons on the extent to which they played a role in the selection of their team captain. Additionally, participants rated their team captain’s leadership quality and level of acceptance. The results consistently indicated that captains who were selected for having good motivational and social competencies were given higher ratings on perceived leadership quality and acceptance by participants. In conclusion, athletes who are motivated, good at motivating others and have superior social skills tend to be better suited for captaincy than those selected based on non-leadership factors.

## Introduction

Leaders have existed since the dawn of human civilization and there are numerous symbols of leadership which can be traced back to nearly 2300 BC. One such symbol is the illustration of a leader, follower and leadership in the ancient Egyptian hieroglyphics (Bass et al., 2008). Since then, interest in the concept of leadership has grown considerably among academics, especially because research has shown that leaders influence followers’ attitudes, motives and behaviors and by doing so, facilitate the group’s success and effectiveness (Hanges et al., 2016). However, what leadership is and how best to practice it continues to be discussed and debated.

Also in sport, researchers have found leadership to be at the heart of optimal team functioning (Cotterill, 2017). Leadership in sport teams manifests itself through both formal and informal roles. Formal leaders, such as coaches and team captains, are those who perform pre-determined leadership responsibilities (Loughead et al., 2006; Gould and Voelker, 2010). Conversely, informal leaders are those who emerge as unofficial leaders, often as a result of natural interactions with other team members (Bucci et al., 2012; Brgoch et al., 2018). Whilst coaches always occupy formal roles, athlete leaders can occupy both formal (e.g., team captain) and informal leadership roles. An emerging body of evidence points toward the vital role of athlete leaders in determining positive team outcomes, including athlete satisfaction (Eys et al., 2007), health and well-being (Fransen et al., 2019b), team cohesion (Loughead et al., 2016), team resilience (Morgan et al., 2015) and team confidence (Fransen et al., 2014a).

Within the present study we focus exclusively on the team captain—the formal athlete leader—who is often expected to perform several essential roles and responsibilities within the team (Newman et al., 2019). Indeed, the team captain serves as the communication bridge between the coach and the players (Camiré, 2016). Moreover, in some sports (e.g., cricket), in conjunction with the coach, the team captain can even be co-responsible for team selection and coordinating tactical decisions both on and off the field (Smith et al., 2018). In addition, the team captain represents their squad during events and meetings to external entities (e.g., media, sponsors, club management etc.; Mosher, 1979). Adding to this list of responsibilities, Dupuis et al. (2006) highlighted the critical role that the team captain plays in supporting their teammates. Furthermore, numerous studies have emphasized that the team captain is expected to provide direct leadership through leading by example and acting as a role model for team members (Dupuis et al., 2006; Cotterill, 2017; Cotterill et al., 2019).

Despite the evidence that the team captain is an important athlete leader within their team, Fransen et al. (2014b) found that only 1% of participants within their study perceived their team captain to be the ‘best’ leader across all four leadership roles (i.e., task, motivational, social and external; for definitions see Table 1). These four leadership roles are derived from early work by Bales and Slater (1955). These researchers distinguished between leaders with an instrumental function, whose primary focus was on accomplishing group tasks, and an expressive function, which was mainly concerned with interpersonal relationships. Influenced by this early research in organizational settings, Rees and Segal (1984) investigated the existence of these roles in sport settings indicating that athlete leaders can fulfill an instrumental and/or an expressive leadership role. In addition to renaming these former leadership roles (i.e., instrumental and expressive) to task and social leadership, Loughead et al. (2006) extended the athlete leadership categorization to include a third role, namely, the external leader. Following this, Fransen et al. (2014b) added the fourth leadership role that is, the motivational leader. These researchers also found that the fulfillment of these four roles (i.e., task, motivational, social and external) resulted in higher team confidence, stronger team identification and better team ranking. However, nearly half (43%) of the 4451 participants (3193 players and 1258 coaches) indicated that the team captain was not the best leader in any of the four leadership roles. These findings remained consistent across gender, team level and sport. A possible explanation for this unexpected outcome, which is of particular relevance to the current study, is that team captains are not always selected for the right reasons (Cotterill et al., 2019; Fransen et al., 2019a).

**TABLE 1:** The definition of the four leadership roles (Fransen et al., 2014b).

**Leadership roles**	**Definition**
Task leader	A task leader is in charge on the field; this person helps his/her team to focus on the team goals and help in tactical decision making. Furthermore, the task leader gives his/her teammates tactical advice during the game and gives them guidance if necessary
Motivational leader	The motivational leader is the biggest motivator on the field; this person encourages teammates to go to any extreme; this leader also put fresh heart into players who are discouraged. In short, this leader steers all the emotions on the field in the right direction in order to maximize team performance.
Social leader	The social leader has a leading role off the field; this person promotes good relations within the team and cares about having a good team atmosphere, for example, in the dressing room, on the bus, or during social activity. Furthermore, this leader helps with conflicts between teammates off the field. He/she is a good listener and is trusted by his/her teammates.
External leader	The external leader is the link between his/her team and the people outside the team; this leader is the representative of the team when dealing with the club management. If communication is needed with media or sponsors, this person will take the lead. This leader will also communicate, the views of the club management to the team, for example, regarding sponsoring, club events, and contracts.

As pioneering researchers who attempted to explicate the reasons for why team captains are selected, Yukelson et al. (1983) observed that captain selection in baseball and football appeared to be based on technical abilities. In line with these findings, Moran and Weiss (2006) found that coaches assigned a higher leadership status to athletes who had superior athletic abilities. Additionally, research by Lee et al. (1983) suggested that team captains were also selected based on their position of play, with football captains more likely to occupy a spatially central playing position compared to their teammates. Furthermore, in many sports (e.g., volleyball, handball, ice hockey etc.), evidence suggests that team captains are likely to be players who occupy positions of high interactional centrality, described as positions that involve a lot of interaction with other players (e.g., midfielder in soccer; Fransen et al., 2016). Contrary to these findings, Tropp and Landers (1979) did not find an association between interactional centrality and team captaincy in collegiate hockey teams. Instead, their findings suggested that team tenure was the discriminating factor between team captains and non-captains, with team captains being those who were the longest serving members of their teams. These findings were recently confirmed and generalized across numerous sports by Fransen et al. (2018) who found that the only characteristic on which team captains outscored the informal athlete leaders was team tenure.

The research on captain selection that we have reviewed above highlights that team captains tend to be athletes who are highly skilled, occupy a central playing position and have a relatively longer team tenure compared to their teammates. However, while these studies provide an indication of the numerous attributes that distinguish team captains from non-captains, they do not directly investigate the reasons for captain selection.

To address this lacuna, Wright and Côté (2003) elaborated on the reasons for captain selection by conducting open-ended interviews with six male athletes competing in basketball, volleyball and ice-hockey. Their results indicated that team captains are often selected for their strong work ethic. Furthermore, Bucci et al. (2012) reported that ice hockey coaches consider the five following psychosocial attributes when appointing team captains: (a) their fit with the team identity, (b) their generosity and honesty, (c) their capacity to lead by example, (d) the common values they share with their teammates and (e) their relationship with the coach. Similarly, Cotterill et al. (2019) interviewed rugby coaches who emphasized that, in addition to leading by example, possessing the trust of one’s teammates is an important attribute for team captain selection. While these studies were more explicit in their interrogation of the reasons underlying captain selection, they were limited in their generalizability given their small sample size.

Overcoming this limitation, Fransen et al. (2019a) used a sample of 226 players and 172 coaches participating in a range of different sports and conducted the most comprehensive study on the reasons underlying captain selection to date. In this study, participants were asked the reasons they perceived to have been used in the selection of their current team captain. These researchers went further by also asking participants to indicate the behaviors, attributes and characteristics of their ideal team captain. The latter investigation helped determine whether the reasons implicated in captain selection match the expectations that players and coaches have of their team captain. The results of this study indicated that non-leadership factors (e.g., being the daughter of the club president, having higher sport specific competence) were perceived to be the primary reasons for captain selection. However, players and coaches indicated that they expect their ideal team captain to have superior motivational skills (e.g., motivating and encouraging team members) and social skills (i.e., having social skills, dealing with conflicts in the team etc.). These findings suggest that there may be a discrepancy between what players and coaches expect of team captains and the criteria based on which these team captains are selected. In turn, this discrepancy might impact team functioning and offers a potential explanation as to why in Fransen et al.’s (2014b) study, team captains were rarely perceived as the ‘best leaders’ across all four leadership roles.

Taken together, there seems to be some initial evidence that team captains are not being selected for the most relevant reasons. However, researchers in this field have failed to associate the reasons implicated in captain selection with captains’ perceived leadership quality (in general and the four leadership roles) and acceptance within the team. These outcome measures are important for three main reasons. First, as argued by Loughead et al. (2006), simply electing and appointing a team captain does little to ensure that the provided leadership is of high quality, effective and fulfills the needs of the team. By unveiling the reasons for captain selection that are associated with high quality leadership, research may be able to shed light on the good and bad practices of captain selection. Second, leadership quality on the four leadership roles (i.e., task, motivational, social and external), as defined and described by Fransen et al. (2014b), may also be important given that their effective fulfillment is associated with higher team confidence, stronger team identification and better team ranking. Moreover, recently too, Fransen et al. (2019a) found that both coaches and players expect their team captain to provide good task, motivational, social and external leadership quality. Therefore, it may be that an athlete who is, on average, good at all leadership roles should be appointed as the team captain. Knowing which reasons for captain selection predict leadership quality on each of these roles may thus be essential information for selectors. Finally, acceptance of the team captain within the team is also an important outcome variable given that previous researchers have demonstrated that the acceptance of the leader by followers facilitates leader effectiveness (e.g., Moran and Weiss, 2006; Price and Weiss, 2011). These findings make sense given that leaders who are not accepted by their team members will tend to have a smaller support base and find it arduous to influence their team members as compared to leaders who are accepted (House et al., 2004; Fransen et al., 2020b). Indeed, leader acceptance has also been proposed as an attribute of an ideal team captain (Fransen et al., 2019a).

Therefore, in order to advance this area of research we investigated the relationships between reasons for which team captains are believed to be selected and (1) the perceived leadership quality of team captains (in general and on the four leadership roles as described by Fransen et al., 2014b); and (2) the perceived acceptance of team captains within their team. Due to the novelty of the research questions, no *a priori* hypotheses were formulated.

## Materials and Methods

### Participants

A total of 653 participants (455 coaches and 198 players) were recruited in Belgium for this study (male = 508, female = 145; football = 439, volleyball = 214), of which 227 competed at the national level and 426 at the regional level. Coaches reported an average age of 45.05 years (*SD* = 11.49) and had on average 15.80 years of experience in their sport. Athletes on the other hand reported an average age of 23.58 years (*SD* = 4.98) and had on average 14.75 years of experience competing in their sport. This study employed convenience and snowball sampling methods. More specifically, participants were recruited via personal contacts, social media forums (e.g., Facebook group of volleyball coaches) and gatekeepers (e.g., Royal Belgian Football Association and Voltraweb). Additionally, we also contacted 25 complete teams of which 20 agreed to participate (response rate = 80%). Utilizing gatekeepers and collecting data from complete teams facilitated in having a diverse participant pool, thus partially limiting self-selection bias persistent across studies employing a similar methodology.

### Procedure

The study was approved by the ethics committee at the university of the first author (G-2020-1728). Prior to starting our quantitative data collection, we assembled 41 reasons which have been and could be used in the selection of the team captain. In doing so, we first scanned previous literature investigating the selection of the team captain. Second, we consulted with football and volleyball coaches and questioned them regarding the reasons they used in the selection of their team captain. Third, to this list, drawing on theory of athlete leadership in sport, we added behaviors typical of task, motivational, social and external leaders (Fransen et al., 2014b) (Table 1). This was done because a number of these behaviors have been implicated in the selection of the team captain and/or associated with ideal team captains (Fransen et al., 2019a). Finally, we also drew upon the identity leadership approach as identity leadership behaviors have shown to be characteristic of high-quality athlete leaders and might be used by selectors during captain appointment (Steffens et al., 2014; Fransen et al., 2020a,c). Our goal was not to provide an exhaustive list but rather to capture the main leadership and non-leadership reasons for captain selection.

The final list of reasons for captain selection was used as the basis for the questionnaire used in this study (see Table 2). In this questionnaire, participants had to indicate their function in the team (coach or player), answer demographic questions (e.g., gender, sport, age, experience, etc.) and respond to the below mentioned measures. The survey took approximately 10–20 min to complete. All participants participated voluntarily and were assured that their data would be treated confidentially. Written informed consent was obtained from all participants. Those who wished to be kept informed were sent an e-mail once the study was complete.

**TABLE 2:** The pattern matrix for the three-component solution.

	**Items**	**Component 1**	**Component 2**	**Component 3**
1.	The team captain displays a lot of effort during the game	0.88		
2.	The team captain has a positive mentality	0.74		
3.	The team captain has a winning mentality	0.74		
4.	The team captain displays a lot of effort during training	0.73		
5.	The team captain is well liked by the team	0.72		
6.	The team captain is concerned about the well-being of his/her teammates	0.69		
7.	The team captain encourages his/her teammates	0.68		
8.	The team captain expresses confidence to his/her teammates	0.67		
9.	The team captain is trusted by the coach	0.67		
10.	The team captain is trusted by his/her teammates	0.64		
11.	The team captain creates a sense of ‘us’ within the team	0.62		0.25
12.	The team captain is helpful	0.57	0.32	
13.	The team captain is the first contact point for his/her teammates	0.56		
14.	The team captain makes sure that there is a good team atmosphere off the field	0.56		
15.	The team captain creates a calm atmosphere within the team	0.55		0.31
16.	The team captain has an excellent insight in the game	0.39		0.36
17.	The team captain scores on average strongest on the different leadership qualities	0.36		0.34
18.	The team captain has a good connection with the coach	0.35	0.33	
19.	The team captain communicates in an efficient way with the referee	0.33		0.26
20.	The team captain is well received among the sponsors		0.83	
21.	The team captain is popular amongst fans		0.83	
22.	The team captain is received well by the board		0.81	
23.	The team captain has been a member of the club for a long time		0.76	–0.21
24.	The team captain is popular with the media		0.75	
25.	The team captain is one of the oldest members within the team		0.72	–0.21
26.	The team captain is able to communicate well with the board		0.72	
27.	The team captain has special ties with the sponsors (family, good acquaintances)	–0.24	0.71	0.28
28.	The team captain has special ties with the board (family, good acquaintances)	–0.22	0.71	0.21
29.	The team captain is respected because of his/her history as a player		0.64	
30.	The team captain has many years of experience within his/her sport	0.24	0.61	
31.	The team captain occupies a central playing position		0.39	
32.	The team captain takes the lead in organizing team activities	0.30	0.38	
33.	The team captain embodies the vision of the club	0.20	0.36	0.35
34.	The team captain has excellent athletic skills	0.26	0.35	
35.	The team captain translates the vision of the coach to the players	0.30		0.67
36.	The team captain clarifies the decisions of the coach when these are not clear for the players			0.64
37.	The team captain is an extension of the coach with respect to providing tactical guidelines on the field			0.64
38.	The team captain defends the coach even if the coach is not present with the team (e.g., in the dressing room)			0.52
39.	The team captain communicates the goals of the team to the players			0.51
40.	The team captain communicates everything that is happening in the team (also what is discussed behind the back of the coach when this is important for the coach)			0.51
41.	The team captain has been chosen by the group of players			0.46

### Measures

#### Reasons Underpinning Captain Selection

Participants were asked to indicate the extent to which they perceived each of the 41 reasons within the final questionnaire to have played a role in the selection of their team captain. We used an 11-point Likert scale ranging from 0 *(strongly disagree)* to 10 *(strongly agree)*.

#### Leadership Quality of the Team Captain

Participants were asked to rate the leadership quality of their team captain in general and on each of the four leadership roles (i.e., task, motivational, social and external) as defined by Fransen et al. (2014b). The definition of each role was provided within the questionnaire (see Table 1). We used an 11-point Likert scale ranging from 0 *(very bad)* to 10 *(very good)*.

#### Acceptance of the Team Captain

Participants were asked to rate the extent to which their team captain was accepted within their team on an 11-point Likert scale ranging from 0 *(not at all)* to 10 *(completely).*

An 11-point Likert scale was used to measure the aforementioned variables with a view to ensure consistency with previous research assessing leadership quality on the four leadership roles (i.e., task, social, motivational and external; Fransen et al., 2019b; Mertens et al., 2020).

### Data Analysis

The data was screened for missing values, outliers and normality. Next, given the exploratory nature of our investigation, participants’ perceived applicability of each of the 41 reasons for appointing the team captain were examined using a principal component analysis (PCA) with direct oblimin rotation. This rotation was adopted because we expected the components to be interrelated. By conducting a PCA, we were able to reduce the large set of reasons to a more manageable set of predictors as well as avoid multi-collinearity within the subsequent analyses (Henson and Roberts, 2006). We determined the minimum number of principal components that accounted for the most amount of variance in our data by assessing (a) the proportion of variance explained; (b) the eigen values; (c) the scree plot; and (d) the component loadings. Some items were not allotted to any one scale because they did not meet the previously established minimum criteria of having a primary loading of 0.55 and no cross-loadings of 0.20 or above (Ford et al., 1986). Scales were built after combining items that loaded on a latent variable. Reliability of these scales were determined by calculating Cronbach’s alphas. Also, descriptive statistics including means and standard deviations for all our study variables were calculated. Moreover, to exclude the possibility of bias in the subsequent analyses, we computed the variance inflation factors (i.e., VIF) for each predictor variable in the regression analyses described below.

To address the first research question (i.e., the relationships between reasons for captain selection and perceived leadership quality of the team captain), the scales and items based on the PCA were used as predictor variables in multiple regressions within which the criterion variable was perceived leadership quality of the team captain as rated by coaches and players (in general as well as on each of the four leadership roles separately). To address the second research question (i.e., the relationships between reasons for captain selection and perceived acceptance of the captain within their team), the predictor variables remained the same while the criterion variable was perceived acceptance of the team captain within the team, as rated by the coaches and players. Age, gender and team level were included as control variables in all the multiple regressions described above.

## Results

### Preliminary Analysis

Missing values accounted for less than 0.6% of the data and were therefore omitted from further analyses (Scheffer, 2002; Van der Heijden et al., 2006). Further, none of the participants in our data set were excluded as outliers and visual inspections of histograms did not reveal any obvious deviations from normality.

The eigen values of the first three components were 12.58, 5.77, and 1.91, and explained 30.70%, 14.09%, and 4.67% of the variance, respectively. The fourth and fifth factors had eigen values just over 1 and explained 4.16% and 3.09% of the total variance. Further, the scree plot suggested that between three and five components should be extracted as the slope precipitously leveled off after this point (Bryant and Yarnold, 1995). Thus, the solutions for three, four and five components were examined.

We opted for the most parsimonious three-component solution. Of the 41 reasons that were subjected to the PCA, nine reasons did not load sufficiently on any one component (above 0.55) and had multiple complex loadings (above 0.20). However, these nine reasons were still included as separate items for further analyses because they could still be important reasons for captain selection and in-turn influence leadership quality and acceptance. Moreover, while allotting items to components we made two exceptions (i.e., items 12 and 18) which deviated slightly from our previously established criterion of considering items part of a component only when it had a cross-loading below 0.20. This was done because these two items had a cross-loading of 0.32 and 0.33 which is only marginally above the previously established cross-loading criterion (component loadings are displayed in Table 2).

Overall, the Cronbach’s alphas were excellent for both the first component, which we labeled ‘Motivational and Social Competencies’ (α = 0.93), and for the second component, which we labeled ‘Representative of the Team’ (α = 0.92). For the third component, which we labeled ‘Extension of the Coach,’ the Cronbach’s alpha was high (α = 0.82). Furthermore, to assist the process of understanding the results it is important to provide an explanation on why labels such as ‘Motivational and Social Competencies,’ ‘Representative of the Team,’ and ‘Extension of the Coach’ were given to these three principal components.

The behaviors that together constituted ‘Motivational and Social Competencies’ (see Table 2) theoretically correspond to descriptions of motivational and social leadership roles as described by Fransen et al. (2014b). However, according to this leadership classification the motivational and social leadership roles are separate and distinct. A potential reason as to why motivational and social leadership behaviors may have loaded upon the same component within the present study may be that they both have a common underlying premise that refers to interpersonal relationships. Indeed, Fransen et al. (2015) found a significant overlap between the leadership quality networks for motivational and social leadership roles. This finding held for male and female teams competing on different levels within football, basketball and volleyball.

What merits discussion here is that the ‘Motivational’ aspect of our principal component ‘Motivational and Social Competencies’ is partially but not completely captured within the definition of motivational leadership by Fransen et al. (2014b). Their definition focuses solely on the behavior of encouraging one’s teammates. Instead, in the present research, the highest loading items on the ‘Motivational and Social Competencies’ (see Table 1) scale indicates that a motivational leader should not only motivate others but must also be motivated themselves (e.g., display a lot of effort during training and games, have a winning and positive mentality). This is in line with previous research that has linked behaviors such as controlling one’s emotions and remaining positive during the game as key behaviors demonstrated by a motivational leader (Dupuis et al., 2006). Furthermore, Fransen et al. (2018) have also identified three characteristics as typical of motivational leaders: being optimistic, exerting high levels of effort during training and using facial expressions or body language that clearly expresses positive emotions. One could argue that the definition of motivational leadership as described within the four-fold leadership classification should be broadened to include the aforementioned aspects (e.g., being motivated).

The second principal component which we labeled ‘Representative of the Team’ is theoretically linked with the external leadership role as defined by Fransen et al. (2014b) (see Table 1). Most behaviors included within this component point toward the integral function of the team captain in representing the team and facilitating communication with external entities (e.g., sponsors, fans and the media; Cotterill, 2017; Brgoch et al., 2018). It should be noted however that items 23, 25, and 30 (see Table 2) fall outside the definition of external leadership. Nevertheless, the placement of these items within this component is not completely unexpected given that external leaders tend to be athletes with the longest team tenure or the oldest player on their team, or both (Loughead et al., 2006; Fransen et al., 2018).

The third principal component labeled ‘Extension of the Coach’ closely aligns with the external leadership role described by Loughead et al. (2006). More specifically, these researchers indicated that representing the team’s interests in meetings with the coaching staff was a behavioral characteristic of external leaders. In line with this description, several other researchers have also highlighted the importance of the role that the team captain plays between the coach and the team (e.g., Mosher, 1979; Dupuis et al., 2006; Camiré, 2016; Cotterill and Cheetham, 2017). Furthermore, coaches in Cotterill et al.’s (2019) study highlighted a somewhat unique aspect of this role by indicating that they view captains as an extension of their authority on the field. Together, these behaviors are represented within the third principal component (i.e., ‘Extension of the Coach’; see Table 1).

Lastly, Table 3 reports the means and standard deviations for all study variables. Besides, all VIF scores were smaller than 3.02 which is below the recommended limit of 10. This means that, multicollinearity issues are not likely to be a cause for concern here (Bowerman and O’connell, 1990; Myers and Myers, 1990).

**TABLE 3:** Means, standard deviations, and correlations between all study variables.

	***M (SD)***	**1**	**2**	**3**	**4**	**5**	**6**	**7**	**8**	**9**	**10**	**11**	**12**	**13**	**14**	**15**	**16**	**17**
1	8.09 *(1.36)*																	
2	7.48 *(1.70)*	0.65**																
3	7.96 *(1.66)*	0.60**	0.65**															
4	7.77 *(1.69)*	0.59**	0.42**	0.48**														
5	6.65 *(2.35)*	0.32**	0.32**	0.22**	0.44**													
6	8.85 *(1.20)*	0.57**	0.41**	0.45**	0.44**	0.21**												
7	8.03 *(1.24)*	0.59**	0.51**	0.63**	0.53**	0.27**	0.38**											
8	5.03 *(2.34)*	0.17**	0.13**	0.09*	0.24**	0.43**	0.08*	0.26**										
9	6.60 *(1.77)*	0.44**	0.49**	0.44**	0.36**	0.31**	0.17**	0.67**	0.28**									
10	7.06 *(2.34)*	0.31**	0.21**	0.29**	0.31**	0.20**	0.19**	0.47**	0.42**	0.43**								
11	7.37 *(2.03)*	0.38**	0.47**	0.35**	0.19**	0.12**	0.22**	0.51**	0.17**	0.48**	0.30**							
12	6.16 *(2.30)*	0.16**	0.24**	0.23**	0.15**	0.10**	0.08*	0.38**	0.40**	0.32**	0.34**	0.42**	.					
13	7.46 *(2.38)*	0.23**	0.15**	0.17**	0.35**	0.36**	0.09*	0.40**	0.41**	0.32**	0.23**	0.06	0.14**					
14	7.14 *(2.12)*	0.33**	0.30**	0.29**	0.27**	0.25**	0.17**	0.47**	0.30**	0.41**	0.26**	0.37**	0.25**	0.22**				
15	6.02 *(3.19)*	0.05	0.12**	0.030	0.04	0.13**	0.050	0.16**	0.38**	0.18**	0.20**	0.28**	0.28**	0.11**	0.20**			
16	6.45 *(2.56)*	0.29**	0.26**	0.22**	0.27**	0.28**	0.13**	0.47**	0.46**	0.49**	0.33**	0.33**	0.36**	0.28**	0.36**	0.38**		
17	7.17 *(2.17)*	0.40**	0.33**	0.37**	0.28**	0.20**	0.23**	0.53**	0.22**	0.49**	0.28**	0.40**	0.29**	0.18**	0.37**	0.12**	0.34**	
18	5.03 *(3.45)*	0.10**	0.15**	0.10*	0.16**	0.21**	0.04	0.16**	0.24**	0.28**	0.14**	0.10**	0.11**	0.20**	0.21**	0.13**	0.24**	0.15**

*p < 0.05; **p < 0.01.

*Abbreviations for the numbers are: General leadership quality of the team captain (1); Leadership quality of the team captain as a task leader (2); Leadership quality of the team captain as a motivational leader (3); Leadership quality of the team captain as a social leader (4); Leadership quality of the team captain as an external leader (5); Acceptance of the team captain within the team (6); Motivational and Social Competencies of the team captain (7); The team captain as a Representative of the Team (8); The team captain as an Extension of the Coach (9); The team captain has a good connection with the coach (10); The team captain has an excellent insight in the game (11); The team captain has excellent athletic skills (12); The team captain takes the lead in organizing team activities (13); The team captain communicates in an efficient way with the referee (14); The team captain occupies a central playing position (15); The team captain embodies the vision of the club (16); The team captain scores on average strongest on the different leadership qualities (17); The team captain has been chosen by the group of players (18).*

### RQ1: The Association Between Reasons for Captain Selection and Perceived Leadership Quality of the Team Captain

Table 4 summarizes the multiple regressions conducted to predict participants’ ratings on general, task, motivational, social and external leadership qualities of the team captain. The primary findings of these regression analyses are presented below.

**TABLE 4:** Linear regressions predicting general, task, motivational, social and external leadership quality as well as acceptance of the team captain.

	***General***	***Task***	***Motivational***	***Social***	***External***	***Acceptance***
	
	***Beta***					
Participants’ age	–0.00	–0.02	0.00	–0.03	–0.03	−0.09*
Participants’ gender	0.00	0.00	0.04	–0.02	0.00	0.00
Participants’ team level	–0.05	–0.01	0.00	–0.01	–0.08	–0.02
Motivational and Social Competencies of the team captain	0.44**	0.27**	0.61**	0.46**	0.02**	0.42**
The team captain as a Representative of the Team	0.05	–0.01	–0.01	0.06	0.36**	0.01
The team captain as an Extension of the Coach	0.00	0.26**	0.04	0.00	0.17	−0.16*
The team captain has a good connection with the coach	0.03	−0.09*	0.01	0.05	–0.06	0.04
The team captain has an excellent insight in the game	0.10*	0.20**	–0.00	−0.11*	–0.01	0.06
The team captain has excellent athletic skills	−0.14*	0.02	0.02	–0.06	−0.11*	−0.10*
The team captain takes the lead in organizing team activities	–0.00	–0.02	–0.07	0.12*	0.15**	–0.05
The team captain communicates in an efficient way with the referee	0.05	0.03	0.00	0.02	0.05	0.00
The team captain occupies a central playing position	−0.07*	0.00	–0.05	–0.06	–0.04	0.00
The team captain embodies the vision of the club	0.02	–0.05	–0.07	0.01	0.01	–0.01
The team captain scores on average strongest on the different leadership qualities	0.10*	–0.00	0.05	0.03	0.01	0.08
The team captain has been chosen by the group of players	–0.02	0.04	0.01	0.04	0.04	0.01
*R*^2^	0.35	0.34	0.40	0.30	0.28	0.16
*F*	21.24**	21.13**	27.13**	18.03**	15.78	7.78**

*p < 0.05; **p < 0.01.

First, with respect to general leadership quality, the results showed that when captains were selected because they had good motivational and social competencies, excellent insights in the game or scored on average strongest on the different leadership qualities, they were perceived as better leaders by participants. In contrast, when captains were selected because they played in a central playing position or had excellent athletic skills, they were perceived as worse leaders by participants.

Second, with respect to task leadership quality, we observed that captains who were selected because they had good motivational and social competencies, acted as an extension of the coach or had excellent insights in the game, were perceived as better task leaders by participants. Contrastingly, if captains were selected because they had a good connection with the coach, they were perceived as worse task leaders by participants.

Third, with respect to motivational leadership quality, we found that captains who were selected based on their motivational and social competencies were perceived as better motivational leaders.

Fourth, with respect to social leadership quality, the results revealed that captains were perceived as better social leaders when they were selected because they had good motivational and social competencies or took the lead in organizing team activities. In contrast, captains were perceived as worse social leaders when they were selected because they had excellent insights in the game.

Finally, with respect to external leadership quality, captains were perceived as better external leaders by participants when they were selected for having good motivational and social competencies, being the representative of the team and taking the lead in organizing team activities. However, captains were perceived as worse external leaders when they were selected because they had excellent athletic skills.

### RQ2: The Association Between Reasons for Captain Selection and Acceptance of the Team Captain Within Their Team

Table 4 summarizes the multiple regression conducted to predict the participants’ perceived acceptance of the team captain within their team. The results revealed that captains were more accepted within their team when they were selected for having good motivational and social competencies. On the contrary, captains were less accepted within their team when they were selected for being an extension of the coach or having excellent athletic skills. Moreover, we also found that older participants tended to accept their team captain to a lesser extent as compared to younger participants.

## Discussion

Researchers before us have provided some answers on why team captains are selected. However, none thus far have investigated the associations between the reasons for captain selection and captains’ perceived leadership quality and level of acceptance, as rated by players and coaches. Therefore, the present research filled an important gap by providing empirical data on such associations. In this, our research provides a more nuanced understanding of the reasons that are important for captain selection.

Our first research question focused on the relationships between the reasons for captain selection and captains’ perceived leadership quality. Here the evidence revealed that motivational and social competencies, as a reason for captain selection, emerged as the strongest and most consistent predictor of perceived leadership quality in general as well as on the four leadership roles (i.e., task, motivational, social and external; Fransen et al., 2014b). This finding corroborates previous research where interpersonal competencies have also been indicated as a decisive factor in determining athlete leadership quality (Holmes et al., 2008; Fransen et al., 2018). For example, Riggio et al. (2003) found that leaders selected on the basis of their superior emotional and social communications skills were evaluated more positively on leadership effectiveness. Moreover, drawing on evidence from organizational research, Polychroniou (2009) investigated the relationship between social skills, personal motivation and transformational leadership. The results of their study revealed that, supervisors’ social skills and personal motivation are positively associated with their leadership ratings. Caruso et al. (2002) have also argued that the quality of leader–follower relationships is dependent upon a leader’s people skills. Thereby, advocating its use whilst leader selection.

In addition to the aforementioned studies, there is a wealth of related research that has highlighted the importance of motivational (Loughead and Hardy, 2005; Paradis and Loughead, 2010) and social (Klonsky, 1991; Mehra et al., 2006; Moran and Weiss, 2006; Price and Weiss, 2011) leadership skills and its influence on leader effectiveness/ratings. Apitzsch (2009) went even further by stating that the absence of a socio-emotional leader (i.e., someone who creates a positive atmosphere on the field) can lead to complete team collapse.

Evidently, the importance of selecting a team captain with good motivational and social competencies has been highlighted within the scientific literature. However, Fransen et al. (2019a) found that, in practice, selectors seldom used it as a reason for captain selection. Instead, participants in their study indicated that the team captain was generally selected based on non-leadership factors. The inconsistency between the attributes that the team captain is expected to embody and the ones based on which they are currently being selected is somewhat concerning. This is because, in the current study, the reasons for captain selection that referred to motivational and social competencies constituted the strongest predictor of task, motivational, social and external leadership quality. This implies that leaders selected on the basis of their superior motivational and social skills are good at performing all four leadership roles, the fulfillment of which has been linked to effective team functioning (Fransen et al., 2017).

A second major finding of the present study with respect to the first research question is that captains were perceived as better leaders in general when they were selected based on their excellent insights in the game. According to the definitions within the four-fold leadership classification, having excellent insights in the game is a behavior that is characteristic of task leaders (Fransen et al., 2014b, 2018). This finding is thus in line with previous research revealing that, effective peer leaders focus on task-related exchanges as well as training and instruction (Murai and Inomata, 2010; Paradis and Loughead, 2010). Furthermore, a study led by Hardy et al. (2008) indicated that task cohesion is higher in teams led by task-oriented peer leaders and task cohesion has been found to be predictive of team performance (Williams and Widmeyer, 1991). In conjunction with our findings, these studies highlight the importance of selecting a leader who has knowledge of their sport (i.e., has excellent insights in the game). However, as was the case with motivational and social competencies, having excellent insights in the game was a factor that was rarely considered by selectors while choosing the team captain (Fransen et al., 2019a).

It is worth noting that, having excellent insights in the game should not be confused with having excellent athletic skills. The former pertains to having knowledge of the sport. For example, knowing where to position players on a field or providing considerable input while developing a game plan. On the other hand, players who are better than their teammates at playing the sport would be considered as having excellent athletic skills. However, it is possible that, players recognized as having excellent athletic skills may not be adept at communicating their sport related knowledge to their teammates and may not be the best leaders.

This leads us to the third major finding of the present study, captains who were selected based on their excellent athletic skills and their central playing position were perceived as worse leaders. This finding is incongruous with previous work on this subject (e.g., Lee et al., 1983; Yukelson et al., 1983; Moran and Weiss, 2006; Fransen et al., 2016). A possible explanation for this discrepancy, as mentioned in the introduction, is that previous research did not explicitly investigate athletic ability and central playing position as reasons for captain selection. Nevertheless, our findings provide further support for Fransen et al.’s (2019a) conclusion that team captains should not be selected based on non-leadership factors such as technical ability and central playing positions, as may currently be the case.

The second research question of this study focused on whether the perceived reasons for captain selection were predictive of the captain’s acceptance by their coach as well as their teammates. Our first finding with respect to this research question was that captains were more accepted within their team when they were selected for having good motivational and social competencies. From previous research in education and sport settings we know that psychosocial skills predict acceptance of the individual by their peers (Moran and Weiss, 2006). Additionally, research on leadership has demonstrated that leader behaviors and attributes predict acceptance of the leader by peers (House and Mitchell, 1975; House et al., 2004; Malik et al., 2014). Taking these findings in conjunction with one another it follows that leaders who possess psychosocial skills may also be more likely to be accepted by their team members as was found in the current study.

Second, our results also showed that captains were less accepted within their team when they were selected for being an extension of the coach. To understand this finding, one could view it through the lens of the identity leadership theory. According to this theoretical perspective, leaders are more effective when they are seen to be acting in ways that serve the interests of their in-group, rather than (a) their personal interests or (b) the interests of other outgroups (Steffens et al., 2014). Consequently, if the team captain is perceived to be acting in the interests of the coach rather than the interests of the players, captains may be seen as out-group members and therefore less accepted by their team members.

Third, the study findings revealed that captains were less accepted within their team when they were selected for having excellent athletic skills. Previous research has suggested that sport specific athletic skills are often used as a criteria underpinning captain selection (Yukelson et al., 1983; Fransen et al., 2019a). In contrast, our research finding suggests that, selecting the team captain based on their excellent athletic skills is not a good approach for creating support from the players in the captain’s leadership. That is, it is not because a player knows how to play the game well that they are also suited to guide other players on and off the field. The findings of the present study thereby undermine the common misperception in the sporting world that the best players should be elected as team captains. Instead, doing so, may reduce the support of the players in the captain’s leadership, and, as a result, the chances of effective leadership may be low.

Finally, we also found that participants who tended to be older accepted the team captain to a lesser extent as compared to participants who were younger. A potential reason for this may be that coaches in our study were considerably older than the players. In light of these findings, it may be that age was a confounding variable with respect to our second research question.

We would like to point out that our study has a number of specific strengths. First, the research questions are novel, and the answers provide a more nuanced understanding of why team captains should be selected. This is important given that researchers have recently acknowledged the lack of clarity regarding criteria that are employed for captain selection and how these relate to captains’ leadership quality and acceptance (Cotterill et al., 2019). Second, this study is amongst the first quantitative studies to employ an extensive list of reasons for captain selection while also using rigorous statistical techniques to collate reasons (i.e., principal component analysis). Third, we were able to recruit relatively large representative samples of both coach and athlete populations in football and volleyball within Flanders.

There are also several limitations of this study. First, the study participants may have rated their perception of captains’ actual behaviors as opposed to those that led to their selection in the first place. A possible way of overcoming this limitation in the future is to conduct research at the start of the season when captains are in the process of being selected. The second limitation of this study is that our results may not be generalizable to other sports. Future researchers should therefore consider replicating this study across different sports, such as cricket and rugby, where the role of the team captain may be more enhanced (Cotterill and Cheetham, 2017). A third limitation is related to the specific culture in which our data were collected. That is, our data collection was limited to coaches and players from Belgium. There is now ample evidence that leadership perceptions and effectiveness – in organizations – is dependent upon context-specific cultural values (House et al., 2004, 2013; Chhokar et al., 2007). For example, participative leadership tends to be preferred within the Germanic regional cluster (including countries such as Germany and the Netherlands). By contrast, countries falling within the Confucian regional cluster (e.g., China, South Korea, etc.) tend to prefer self-protective forms of leadership (House et al., 2004). It is very likely then that cross-cultural differences in leadership preferences are also prevalent within sporting contexts which in-turn may influence the reasons for captain selection and its association with leadership quality and acceptance. Therefore, it would be interesting to see whether our findings hold across national borders. A fourth limitation is that we used a single item format to assess leadership quality (both in general as well as on the four leadership roles) and acceptance of the team captain. However, it could be argued that these items were relatively straightforward and therefore sufficient to measure using a single item. Additionally, single item measures offer practical advantages such as shortened survey length and reduced research costs. Statistically too, they reduce the chance of encountering common method variance while also adding to a construct’s face validity (Hoeppner et al., 2011).

There are also several avenues for future research. First, researchers could control for additional variables including the number of years the team captain has been involved in the sport, the number of years the team captain has led their sport team, the role (e.g., on-field leader) the team captain is expected to play within their sport team etc. Second, future researchers can explore the relationships between reasons for captain selection, leadership quality, leader acceptance and the impact of those on team effectiveness measures (e.g., cohesion, health and well-being, collective efficacy etc.). These team effectiveness measures should also include objective variables including team ranking, the amount of revenue the team captain brings in for the club or the sport team etc. Finally, another opportunity for future research pertains to developing the leadership competencies of team captains as researchers have argued that identifying the right leader is only the first step (Fransen et al., 2015). While previous researchers have already provided an insight into potential leadership programs that could support the process of developing leadership competencies (Gould et al., 2013; Cotterill, 2017; Newman et al., 2019; Fransen et al., 2020b), the present research provides an understanding into how these programs can be improved further. More specifically, based on our findings, it would be of interest to explore interventions that exclusively target developing and training motivational and social skills of team captains.

We also see a number of practical implications of our findings. First and foremost, we suggest that selectors should choose team captains based predominantly on their motivational and social skills (i.e., interpersonal skills). Furthermore, based on the results of this study, other pertinent reasons for captain selection that should be taken into consideration depending on the needs of the team are having excellent insights in the game, being a representative of the team and taking the lead in organizing team activities. For example, in a season when a team may require more task-related guidance, selectors might appoint a team captain who has excellent insights in the game (i.e., task leadership qualities) in addition to having good interpersonal skills. In order to make this selection, coaches and club management might consider making use of a tool named Shared Leadership Mapping (Fransen et al., 2020b). This tool relies on a technique known as Social Network Analysis and can help selectors identify key leaders in the team with respect to different leadership roles (e.g., task, motivational, social and external). Moreover, Social Network Analysis is grounded in the perception of players rather than that of selectors. It therefore increases the likelihood that the identified leader will be accepted by team members, thus, maximizing the leader’s effectiveness. Second, based on the study findings, we also recommend that selectors do not appoint team captains by virtue of central playing position and superior athletic skill. Finally, we emphasize that in order for team captains to be considered high-quality leaders (in general as well as on the four leadership roles) and to be accepted by their team members, they need to express a wide range of leadership behaviors ranging from task-related guidance to good interpersonal skills. This begs the question, does the expertise required from team captains today exceed the potential of a single individual? Indeed, as we mentioned in the introduction, Fransen et al. (2014b) found that only 1% of team captains were seen as the ‘best’ leader on all four leadership roles. Therefore, in addition to selecting a team captain with superior leadership skills, coaches and club management should consider selecting a leadership team consisting of multiple leaders performing different roles and responsibilities. Indeed, research on shared leadership has grown in the last decade as its benefits have become more apparent within the sport psychology literature (Fransen et al., 2017).

## Conclusion

Considering that the team captain is an important member of a sport team, this position should be awarded with care to those who are motivated, good at motivating others and have good social skills. Moreover, selectors should refrain from selecting a team captain merely based on a player’s central playing position and/or superior athletic skill. Also, this study extends previous research insofar as it provides more quantitative evidence regarding criteria that should be employed when a team has specific leadership needs (e.g., task, external etc.). We hope that this study serves as a step in the direction of filling the gap highlighted by Cotterill et al. (2019), who called for the development of specific evidence-based approaches to captain selection.

## Data Availability Statement

The raw data supporting the conclusions of this article will be made available by the authors, without undue reservation.

## Ethics Statement

This study involving human participants was reviewed and approved by Social and Societal Ethics Committee (SMEC) KU Leuven. The participants provided their written informed consent to participate in this study.

## Author Contributions

JL, KF, and FB conceived the idea and designed this research project. JL collected the data which was analyzed by RB with the help of FB, KF, and PC. This research manuscript was written by RB under the supervision of FB, KF, and PC. All authors contributed to the article and approved the submitted version.

## Conflict of Interest

The authors declare that the research was conducted in the absence of any commercial or financial relationships that could be construed as a potential conflict of interest.
