# Supplementary material for: Why the Chosen Ones May Not Always Be the Best Leaders: Criteria for Captain Selection as Predictors of Leadership Quality and Acceptance
Source: Front Psychol. 2021 Jan 18;11:616966. doi: 10.3389/fpsyg.2020.616966 (PMC7848118; doi:10.3389/fpsyg.2020.616966)
Supplement: Supplementary file 1 [file Data_Sheet_1.PDF]

Supplementary materials:

Linear regressions for different samples

*Table 1: Linear regressions predicting the **general** leadership quality of the team captain*

	Coaches	*Players*	*Male*	*Female*	*Football*	*Volleyball*
				Beta		
Age	.05	.01	.01	-.08	-.02	.07
Gender	.04	.00			-.06	-.05
Team Level	-.08*	-.00	-.07	-.05	-.03	-.02
Motivational and social competencies	.42**	.47**	.40**	.51**	.42**	.52**
Representative of the team	.04	-.09	.09	-.09	.08	-.06
Extension of the Coach	.03	.05	.04	-.09	.06	-.12
This player has a good connection with the coach	.07	-.03	.03	.03	.06	-.02
This player has an excellent insight in the game	.12*	.03	.12*	.00	.13*	.04
This player has excellent athletic skills	-.13*	-.14*	-.13*	-.17	-.19**	-.01
This player takes the lead in organizing team activities	- .07	.16*	-.01	-.01	-.01	.00
This player communicates in an efficient way with the referee	.05	.08	.05	.00	.00	.14*
This player has a central playing position on the field	-.10*	.01	-.08*	.00	-.06	-.11
This player embodies the vision of the club	.06	-.09	.07	-.06	.05	.05
This player scores on average strongest on the different leadership qualities	.07	.19*	.09*	.15	.08	.09
This player has (also) been chosen by the group of players	-.04	.02	-.08*	.14	-.05	.02
R^2	.36	.41	.39	.32	.38	.33
F	16.0**	7.58**	21.72**	3.92**	16.74**	5.92**

*p < .05 **p <0.01

*Table 2: Linear regressions predicting the **task** leadership quality of the team captain*

	Coaches	*Players*	*Male*	*Female*	*Football*	*Volleyball*
			Beta			
Age	.05	-.09	-.00	-.10	-.03	.04
Gender	.01	-.10			.00	-.05
Team Level	.00	.11	.00	-.06	-.04	-.05
Motivational and social competencies	.18**	.39**	.15*	.49**	.22*	.37**
Representative of the team	-.09	-.09	-.00	-.16	-.02	-.11
Extension of the Coach	.33**	.15	.34**	-.02	.34**	.11
This player has a good connection with the coach	-.06	-.13	-.09*	-.08	-.01	-.22*
This player has an excellent insight in the game	.21**	.14	.22**	.04	.24**	.17*
This player has excellent athletic skills	.05	-.03	.02	.04	-.04	.10
This player takes the lead in organizing team activities	-.05	.04	-.03	.01	-.00	-.02
This player communicates in an efficient way with the referee	.08	-.04	.09*	-.17*	-.01	.12
This player has a central playing position on the field	.04	-.02	.02	.00	-.00	.00
This player embodies the vision of the club	.00	.16	.00	-.12	.02	-.06
This player scores on average strongest on the different leadership qualities	-.06	.21*	-.04	.20*	-.12*	.21*
This player has (also) been chosen by the group of players	.01	.13*	-.01	.19*	.02	.01
R^2	.37	.41	.39	.40	.37	.43
F	17.03**	8.25**	21.85**	5.76**	16.02**	9.46**

*p < .05 **p <0.01

*Table 3: Linear regressions predicting the **motivational** leadership quality of the team captain*

	Coaches	*Players*	*Male*	*Female*	*Football*	*Volleyball*
			Beta			
Age	.05	-.00	.01	-.05	.00	.03
Gender	-.00	-.00			.01	.02
Team Level	.03	.09	-.02	-.01	-.05	.06
Motivational and social competencies	.52**	.71**	.54**	.72**	.59**	.63**
Representative of the team	-.06	-.10	.01	-.18	-.03	.03
Extension of the Coach	.04	.14	.05	.05	.06	-.03
This player has a good connection with the coach	.06	-.09	.04	-.09	.04	-.04
This player has an excellent insight in the game	.00	-.00	.01	-.01	.02	.03
This player has excellent athletic skills	.10*	-.12*	.05	-.10	-.00	.05
This player takes the lead in organizing team activities	-.10*	.03	-.09*	-.02	-.09*	.01
This player communicates in an efficient way with the referee	.04	-.02	.05	-.12	-.01	.08
This player has a central playing position on the field	-.06	-.01	-.07	.08	-.01	-.14*
This player embodies the vision of the club	-.03	-.16	-.03	-.13	-.06	-.06
This player scores on average strongest on the different leadership qualities	.05	.09	.05	.06	.04	.08
This player has (also) been chosen by the group of players	.00	.02	-.02	.05	.01	-.03
R^2	.38	.51	.41	7.56	.39	.46
F	17.53**	12.27**	23.62**	.46**	17.86**	10.90**

*p < .05 **p <0.01

*Table 4: Linear regressions predicting the **social** leadership quality of the team captain*

	Coaches	*Players*	*Male*	*Female*	*Football*	*Volleyball*
				Beta		
Age	-.00	-.02	-.00	-.09	-.07	.09
Gender	-.00	-.08			-.00	-.04
Team Level	.02	.03	-.01	-.06	-.05	-.07
Motivational and social competencies	.35**	.62**	.35**	.76**	.42**	.52**
Representative of the team	.07	-.13	.07	-.03	.08	-.04
Extension of the Coach	.03	.00	.07	-.16	.03	-.09
This player has a good connection with the coach	.07	-.04	.06	-.04	.09	-.03
This player has an excellent insight in the game	-.11*	-.07	-.09	-.22*	-.05	-.19*
This player has excellent athletic skills	-.01	-.13*	-.03	-.10	-.06	-.03
This player takes the lead in organizing team activities	.14*	.11	.15*	.00	.12*	.14*
This player communicates in an efficient way with the referee	.00	.08	.01	.01	-.01	.10
This player has a central playing position on the field	-.09*	.00	-.07	-.04	-.06	-.10
This player embodies the vision of the club	.08	-.18*	.07	-.09	-.00	.13
This player scores on average strongest on the different leadership qualities	.02	.15*	.02	.09	-.00	.12
This player has (also) been chosen by the group of players	.04	.05	.05	-.00	.04	.04
R^2	.30	.45	.32	.43	.29	.39
F	12.20**	9.40**	16.22**	6.59**	11.55**	8.21**

*p < .05 **p <0.01

*Table 5: Linear regressions predicting the **external** leadership quality of the team captain*

	Coaches	*Players*	*Male*	*Female*	*Football*	*Volleyball*
				Beta		
Age	-.02	-.05	-.01	-.20*	-.00	-.13
Gender	-.04	-.21			-.07	-.08
Team Level	.03	.08	-.04	-.13	-.03	.12
Motivational and social competencies	-.04	.21*	-.00	.16	.01	.05
Representative of the team	.33**	.18	.37**	.23*	.34**	.36**
Extension of the Coach	.19*	.14	.14*	.24*	.19*	.15
This player has a good connection with the coach	-.06	-.04	-.07	.01	-.02	-.14
This player has an excellent insight in the game	-.02	-.00	.00	-.07	-.02	-.01
This player has excellent athletic skills	-.11*	-.09	-.13*	.03	-.10*	-.09
This player takes the lead in organizing team activities	.16*	.16*	.13*	.25*	.19**	.08
This player communicates in an efficient way with the referee	.08	-.04	.08	-.08	.02	.09
This player has a central playing position on the field	-.04	-.00	-.02	-.07	-.02	.12
This player embodies the vision of the club	.06	-.07	.06	-.11	-.02	.12
This player scores on average strongest on the different leadership qualities	.05	-.08	.04	-.16	.01	.02
This player has (also) been chosen by the group of players	.00	.16*	.01	.09	.03	.07
R^2	.28	.31	.28	.38	.29	.28
F	11.02**	5.28**	13.75**	5.36**	11.27**	5.02**

*p < .05 **p <0.01

Table 6: Linear regressions predicting the ***acceptance*** *of the team captain*

	Coaches	*Players*	*Male*	*Female*	*Football*	*Volleyball*
			Beta			
Age	-.02	-.17*	-.03	-.25*	-.12*	-.00
Gender	.04	.02			.00	-.11
Team Level	-.03	.02	-.03	-.06	-.03	.03
Motivational and social competencies	.40**	.44**	.39**	.53**	.43**	.40**
Representative of the team	.01	-.06	.05	-.18	.01	-.11
Extension of the Coach	-.14*	-.16	-.13*	-.20	-.13*	-.24*
This player has a good connection with the coach	.06	.03	.07	-.05	.09	-.08
This player has an excellent insight in the game	.09	-.01	.11*	-.10	.11	-.02
This player has excellent athletic skills	-.09	-.09	-.10*	-.10	-.08	-.10
This player takes the lead in organizing team activities	-.09	-.12	-.06	-.01	-.07	-.00
This player communicates in an efficient way with the referee	-.00	.02	-.02	-.00	-.07	.09
This player has a central playing position on the field	-.05	.17*	-.03	.18	.00	.01
This player embodies the vision of the club	.01	-.12	-.00	.00	-.08	.17
This player scores on average strongest on the different leadership qualities	.07	.13	.09	.05	.09	.12
This player has (also) been chosen by the group of players	-.00	.09	.02	.02	.06	-.04
R^2	.15	.25	.17	.25	.20	.19
F	5.10**	3.99**	7.18**	2.89**	6.85**	2.91**
